# Correlation analysis between serum uric acid and carotid intima-media thickness: a cross sectional study

**DOI:** 10.3389/fendo.2025.1506964

**Published:** 2025-04-24

**Authors:** Ziheng Zhang, Peng Zhang, Xiangli Yu, Zhongmin Ji, Aimei Zhang, Hongjun Wang, Daojing Li

**Affiliations:** ^1^ Clinical Medical College, Jining Medical University, Jining, China; ^2^ Department of Neurology, Jining First People’s Hospital, Jining, China; ^3^ Department of Neurology, Affiliated Hospital of Jining Medical University, Jining, China; ^4^ Department of Medical Imaging, Affiliated Hospital of Jining Medical University, Jining, China

**Keywords:** intima media thickness, uric acid, carotid atherosclerosis, cerebrovascular disease, cross sectional study

## Abstract

**Objective:**

This study aims to investigate the association between serum uric acid (UA) and carotid intima-media thickness (CIMT) in adults undergoing routine health screenings.

**Methods:**

Clinical data from 375 participants (mean age: 64.26 ± 9.97 years; 48.53% male) who underwent health examinations at Jining Medical University Affiliated Hospital (January 2022–January 2023) were analyzed. ​Generalized additive models and piecewise linear regression were used to evaluate linear/non-linear relationships and threshold effects.

**Results:**

The study included a total of 375 individuals, with an average age of 64.26 ± 9.97 years. The participants consisted of 48.53% males. After adjusting for confounding factors (age, sex, BMI, etc.), a non-linear relationship between UA and CIMT was identified. The threshold occurred at UA = 3.15 mg/dL. ​When UA ≥ 3.15 mg/dL, each 1 mg/dL increase in UA was associated with a 0.061 mm increase in CIMT (β = 0.061, 95% CI: 0.031–0.090, p < 0.0001). No significant association was observed when UA < 3.15 mg/dL (β = −0.002, 95% CI: −0.033–0.030, p = 0.9240).

**Conclusion:**

The study demonstrates a non-linear relationship between UA and CIMT in the health screening population. UA levels ≥3.15 mg/dL are positively correlated with increased CIMT, suggesting that elevated UA may promote carotid atherosclerosis progression.

## 1 Introduction

The global expansion of the elderly population ​has been accompanied by a rising incidence of stroke. Despite significant advancements in stroke treatment and prevention strategies, ​stroke-related mortality and disability rates remain persistently high (1). Atherosclerotic plaques, ​a predominant precursor of ischemic stroke, predominantly occur in the internal carotid artery distal to the bifurcation of the common carotid artery in Western populations. ​This anatomical predilection may be associated with reduced shear stress at this arterial segment. ​Compromised endothelial function, characterized by increased intimal thickness and diminished nitric oxide release under low shear stress conditions, ​contributes to the susceptibility to cholesterol plaque formation. ​Since 2015, stroke ​has emerged as the leading cause of mortality and disability in China, posing substantial threats to public health and socioeconomic stability (2).

Atherosclerosis serves as the principal pathological foundation for cardiovascular and cerebrovascular diseases (3, 4). Carotid intima-media thickness (CIMT), ​a non-invasive ultrasonographic marker, provides reliable assessment of subclinical atherosclerosis and endothelial dysfunction (5, 6). Growing evidence supports CIMT ​as a predictive biomarker for cardiovascular events and stroke (7–9). While traditional risk factors for atherosclerosis ​are well-characterized, ​the pathophysiological contributions of certain metabolic parameters ​require further elucidation.

Urine is the main route for excreting serum uric acid (UA), which is the final byproduct of purine metabolism synthesized in the liver (10).The elevation of UA concentration in human plasma is influenced by factors such as diet, alcohol consumption, fructose intake, obesity, and ethnicity (11).Many studies have demonstrated that an excess of uric acid can lead to conditions such as gout, kidney stones, and inflammatory reactions (12–15).There is an increasing body of evidence supporting the promotive role of UA in atherosclerosis. However, it is noteworthy that some studies have indicated its antioxidative effects in oxidative stress (16), suggesting a protective role for blood vessels in the human body (17, 18). Contradictory data (19–21) characterizes the involvement of UA in the development of atherosclerosis. Furthermore, there is no consensus on the optimal UA level control in healthy populations to manage atherosclerosis. Hence, it is crucial to further explore the connection between UA and CIMT in people undergoing health examinations, providing a basis for future medication interventions that aim to regulate UA levels in order to prevent the onset and progression of atherosclerosis.

## 2 Materials and methods

### 2.1 Study population

Data from 560 individuals who were undergoing health check-ups at the Health Check Center of the Affiliated Hospital of Jining Medical College between January 2022 and January 2023 were analyzed in this cross-sectional study. Inclusion criteria: (1) age ≥ 18 years and (2) signed informed consent. Exclusion criteria: (1) a history of gout or the use of medications affecting uric acid metabolism, (2) significant organ failure in the heart, kidneys, lungs, liver, etc., (3) comorbidities like tumors, rheumatic diseases, or autoimmune diseases, and (4) incomplete data collection for UA and carotid ultrasound. Participants with gout (n = 23), tumors or autoimmune diseases (n = 13), incomplete carotid ultrasound data (n = 86), or missing UA data (n = 63) were excluded from the analysis. The final analysis included 375 participants. Health screenings involved a comprehensive assessment, including UA levels, carotid ultrasound, and other laboratory tests.

### 2.2 General information

The hospital’s health examination system provided comprehensive details about the participants, encompassing their gender, age, systolic blood pressure, diastolic blood pressure, BMI, blood creatinine, fasting blood sugar, triglycerides, total cholesterol, low-density lipoprotein cholesterol, high-density lipoprotein cholesterol, homocysteine, neutrophil count, platelet count, albumin, medical history (including diabetes, hypertension, coronary heart disease, and stroke), usage of antiplatelet and statin medications, smoking habits, and alcohol consumption. Blood pressure was measured following the American College of Cardiology (ACC) guidelines.

### 2.3 Laboratory measurements

After fasting for 8-12 hours, fasting blood samples were collected for laboratory analysis. An automated biochemical analyzer (Cobas) was used to measure UA levels, TC, HDL-C, LDL-C, TG, and FPG. The concentration of glycated hemoglobin (HbA1c) was determined through high-performance liquid chromatography, while the measurement of plasma glucose was conducted using the hexokinase method.

### 2.4 Ultrasound image analysis

Carotid ultrasound examinations were conducted using a portable LOGIQ ultrasound machine (GE, Best, USA). Trained and certified ultrasound physicians followed standard scanning and reading protocols. CIMT measurements were obtained at six different positions near the division point of the common carotid artery, 1 cm above and below the division point on both sides. To enhance reliability and eliminate measurement errors, each of these six locations was measured twice, and the values were averaged.

### 2.5 Statistical analysis

Statistical analyses were conducted using Empower Stats and R software version 4.2.0. Descriptive statistics were employed for general information and biochemical variables. Mean (standard deviation) was used to express continuous variables with a normal distribution, whereas the median was used for non-normally distributed continuous variables. Frequencies or percentages were used to present categorical variables. Univariate analysis models were used to examine the correlation of UA and other anthropometric and biochemical variables with CIMT. Following the adjustment for possible confounding variables, a sleek curve fitting technique was utilized to investigate the correlation between UA and CIMT. Multivariate segmented linear regression models were further employed to assess the independent correlation between UA and CIMT based on the smooth curve fit. Threshold effect analysis was used to determine the presence of inflection points. A significance level of less than 0.05 was attributed to the p-value.

## 3 Results

### 3.1 Participant characteristics

There are a total of 375 individuals involved in the study, consisting of 182 (46.75%) males and 193 (53.25%) females. Participants had an average age of 64.264 ± 9.974 years. The mean levels of UA and CIMT were 3.104 ± 0.781 mg/dL and 0.763 ± 0.123 mm, respectively. For UA, the median values ranged from 1.298 to 5.148 mg/dL, while for CIMT, it ranged from 0.5 to 1.095 mm. In order to investigate the connection between UA and CIMT, UA was divided into four groups (Q1-Q4) according to quartiles, and the initial characteristics of this group are described in **Table 1**.

**Table 1:** Comparison of demographic, clinical, and laboratory characteristics in groups. (n=375).

UA Category	Q1 (N=93) 1.298-2.552	Q2 (N=94) 2.553-3.014	Q3 (N=91) 3.015-3.590	Q4 (N=97) 3.602-5.148	P-value
Age, y	64.04 ± 10.34	65.80 ± 9.60	63.88 ± 10.70	63.35 ± 9.23	0.270
SEX (%)					<0.001
Male (%)	25 (26.88%)	38 (40.43%)	56 (61.54%)	63 (64.95%)	
Female (%)	68 (73.12%)	56 (59.57%)	35 (38.46%)	34 (35.05%)	
SBP, mmHg	134.06 ± 18.78	135.49 ± 19.91	133.21 ± 15.38	135.59 ± 18.49	0.697
DBP, mmHg	77.53 ± 12.28	78.61 ± 12.47	80.56 ± 11.17	80.87 ± 11.56	0.142
Heart rate, beats per minute	74.903 ± 9.537	74.691 ± 11.746	73.736 ± 11.326	76.237 ± 13.212	0.522
BMI, kg/m²	24.30 ± 2.85	24.31 ± 3.54	24.99 ± 3.28	26.30 ± 2.95	<0.001
CR, µmol/L	55.88 ± 16.99	60.12 ± 13.69	63.48 ± 14.72	72.60 ± 24.63	<0.001
Cystatin C, mg/L	1.01 ± 0.22	1.11 ± 0.45	1.12 ± 0.34	1.15 ± 0.25	<0.001
FPG, mmol/L	5.57 ± 2.00	5.31 ± 1.75	5.10 ± 1.01	5.53 ± 1.91	0.289
Triglyceride, mmol/L	1.24 ± 0.66	1.32 ± 0.70	1.34 ± 0.64	1.66 ± 1.13	0.006
Total cholesterol, mmol/L	4.22 ± 0.98	4.15 ± 1.21	4.02 ± 0.88	4.25 ± 1.16	0.424
HDL-C, mmol/L	1.31 ± 0.28	1.31 ± 0.35	1.24 ± 0.25	1.18 ± 0.28	0.004
LDL-C, mmol/L	2.55 ± 0.79	2.44 ± 0.93	2.43 ± 0.77	2.71 ± 0.87	0.112
HCY, µmol/L	1.24 ± 0.66	1.32 ± 0.70	1.34 ± 0.64	1.66 ± 1.13	0.006
Neutrophil count,10^9/L	3.46 ± 1.16	3.53 ± 1.46	3.42 ± 1.01	3.67 ± 1.11	0.192
TLC,10^9/L	1.87 ± 0.52	1.90 ± 0.63	2.06 ± 0.66	2.04 ± 0.55	0.117
Platelet count,10^9/L	231.91 ± 52.60	219.55 ± 62.63	227.45 ± 52.21	226.16 ± 53.79	0.691
Albumin, g/L	40.22 ± 3.17	40.80 ± 4.52	41.15 ± 4.65	42.01 ± 3.36	0.010
Diabetes mellitus (%)					0.329
0	70 (75.27%)	73 (77.66%)	78 (85.71%)	78 (80.41%)	
1	23 (24.73%)	21 (22.34%)	13 (14.29%)	19 (19.59%)	
Hypertension (%)					0.073
0	47 (50.54%)	45 (47.87%)	40 (43.96%)	32 (32.99%)	
1	46 (49.46%)	49 (52.13%)	51 (56.04%)	65 (67.01%)	
CVD (%)					0.301
0	74 (79.57%)	64 (68.09%)	64 (70.33%)	68 (70.10%)	
1	19 (20.43%)	30 (31.91%)	27 (29.67%)	29 (29.90%)	
Stroke (%)					0.186
0	82 (88.17%)	74 (78.72%)	73 (80.22%)	74 (76.29%)	
1	11 (11.83%)	20 (21.28%)	18 (19.78%)	23 (23.71%)	
Antiplatelet drug use (%)					0.048
0	77 (82.80%)	66 (70.21%)	59 (64.84%)	69 (71.13%)	
1	16 (17.20%)	28 (29.79%)	32 (35.16%)	28 (28.87%)	
Statins use (%)					0.221
0	78 (83.87%)	67 (71.28%)	68 (74.73%)	74 (76.29%)	
1	15 (16.13%)	27 (28.72%)	23 (25.27%)	23 (23.71%)	
Smoking status (%)					<0.001
0	84 (90.32%)	72 (76.60%)	61 (67.03%)	59 (60.82%)	
1	9 (9.68%)	22 (23.40%)	30 (32.97%)	38 (39.18%)	
Alcohol consumption status (%)					0.001
0	83 (89.25%)	77 (81.91%)	64 (70.33%)	66 (68.04%)	
1	10 (10.75%)	17 (18.09%)	27 (29.67%)	31 (31.96%)	

Data are presented as mean ± standard deviation or n (%).

BMI, body mass index; CIMT, carotid intima media thickness; Cr, creatinine; CVD, Cardiovascular Disease; DBP, diastolic blood pressure; FPG, fasting plasma glucose; HCY, plasma homocysteine; HDL-C, high-density lipoprotein cholesterol; LDL-C, low-density lipoprotein cholesterol; SBP, systolic blood pressure; TC, total cholesterol; TLC, total lymphocyte count; TG, triglyceride; UA, uric acid. “0” means no, “1” means yes.

### 3.2 Univariate analysis

To investigate the correlation between clinical parameters and CIMT, a single-variable linear regression analysis was conducted. **Table 2** shows a noteworthy correlation between UA and CIMT, indicating a positive relationship.UA was categorized into four groups based on quartiles, revealing statistically significant associations in the Q3 and Q4 groups (p = 0.02623, p ≤ 0.00001). CIMT exhibited no significant correlation with BMI, triglycerides, and medical history (p > 0.05).

**Table 2:** Univariate Analysis of CIMT.

Covariate	Statistics	Effect size	P-value
Age, y	64.264 ± 9.974	0.003 (0.002, 0.004)	<0.00001
Sex, male	182 (48.533%)	Ref	
Sex, female	193 (51.467%)	-0.061 (-0.085, -0.036)	<0.00001
SBP, mmHg	134.608 ± 18.193	0.000 (-0.000, 0.001)	0.47153
DBP, mmHg	79.397 ± 11.917	-0.001 (-0.002, -0.000)	0.04462
BMI, kg/m²	24.986 ± 3.259	0.000 (-0.004, 0.004)	0.97703
BMI category, kg/m²			
<24	146 (38.933%)	Ref	
≥24, <28	162 (43.200%)	-0.021 (-0.049, 0.006)	0.13068
≥28	67 (17.867%)	-0.002 (-0.038, 0.034)	0.91437
UA, mg/dL	3.104 ± 0.781	0.037 (0.021, 0.052)	<0.00001
UA category, mg/dL			
Q1	93 (24.800%)	Ref	
Q2	94 (25.067%)	0.031 (-0.004, 0.065)	0.08288
Q3	91 (24.267%)	0.040 (0.005, 0.074)	0.02623
Q4	97 (25.867%)	0.081 (0.047, 0.115)	<0.00001
Neutrophil count,10^9/L	3.523 ± 1.198	0.012 (0.002, 0.022)	0.02213
Cystatin C, mg/L	1.099 ± 0.329	0.057 (0.019, 0.095)	0.00333
TLC,10^9/L	1.967 ± 0.596	0.002 (-0.019, 0.023)	0.81933
Platelet count,10^9/L	226.245 ± 55.445	-0.000 (-0.000, 0.000)	0.38947
FPG, mmol/L	5.380 ± 1.724	0.002 (-0.005, 0.010)	0.50670
Albumin, g/L	41.056 ± 4.008	-0.003 (-0.007, -0.000)	0.02796
Triglyceride, mmol/L	1.393 ± 0.825	0.003 (-0.012, 0.018)	0.68291
HDL, mmol/L	1.258 ± 0.297	-0.054 (-0.096, -0.013)	0.01079
LDL, mmol/L	2.533 ± 0.848	-0.020 (-0.034, -0.005)	0.00754
HCY, µmol/L	11.289 ± 4.662	0.006 (0.003, 0.008)	0.00006
Hypertension (%)			
0	164 (43.733%)	Ref	
1	211 (56.267%)	0.045 (0.020, 0.070)	0.00042
Diabetes mellitus (%)			
0	299 (79.733%)	Ref	
1	76 (20.267%)	0.010 (-0.021, 0.041)	0.51582
Stroke (%)			
0	303 (80.800%)	Ref	
1	72 (19.200%)	0.013 (-0.018, 0.045)	0.40582
CVD (%)			
0	270 (72.000%)	Ref	
1	105 (28.000%)	0.012 (-0.016, 0.039)	0.40916
Antiplatelet drug use (%)			
0	271 (72.267%)	Ref	
1	104 (27.733%)	0.047 (0.020, 0.075)	0.00084
Statins drug use (%)			
0	287 (76.533%)	Ref	
1	88 (23.467%)	0.036 (0.007, 0.066)	0.01532
Smoking status (%)			
0	276 (73.600%)	Ref	
1	99 (26.400%)	0.048 (0.020, 0.076)	0.00090
Alcohol consumption status (%)			
0	290 (77.333%)	0	
1	85 (22.667%)	0.036 (0.006, 0.066)	0.01739

BMI, body mass index; CIMT, carotid intima media thickness; Cr, creatinine; CVD, Cardiovascular Disease; DBP, diastolic blood pressure; FPG, fasting plasma glucose; HCY, plasma homocysteine; HDL-C, high-density lipoprotein cholesterol; LDL-C, low-density lipoprotein cholesterol; SBP, systolic blood pressure; TC, total cholesterol; TLC, total lymphocyte count; TG, triglyceride; UA, uric acid. “0” means no, “1” means yes; Ref, reference.

### 3.3 Linear regression results of UA and CIMT

In the original model, every 1 mg/dL rise in UA was associated with a 0.037 mm increase in CIMT (β=0.037; 95% CI=0.021–0.052, p<0.00001).After making minimal adjustments for age, sex, and BMI, the model still showed a noteworthy association (β=0.031; 95% CI=0.015-0.048, p=0.00017).After controlling for relevant confounding factors such as sex, age, BMI, HCY, CR, HBP, CYC, antiplatelet drug use, smoking status, alcohol consumption status, DLP, HDL, LDL, NEU, ALB, DBP, and removing factors with Variance Inflation Factors greater than 10 from the fully adjusted model, the adjusted model II still demonstrated a significant positive linear association between UA and CIMT (β=0.033; 95% CI=0.015-0.050, p=0.00032).However, a statistically significant association between UA and CIMT was observed only in Q4 when grouped by quartiles (β=0.069; 95% CI=0.031-0.106, p=0.00036).This suggests a potential non-linear relationship between UA and CIMT (**Table 3**).

**Table 3:** Relationship between UA and CIMT in Different Models.

Variable	Crude model	Minimally adjusted model	Fully adjusted model
UA	0.037 (0.021, 0.052) <0.00001	0.031 (0.015, 0.048) 0.00017	0.034 (0.016, 0.051) 0.00028
UA(%) category			
Q1	Ref	Ref	Ref
Q2	0.031 (-0.004, 0.065) 0.08288	0.019 (-0.014, 0.052) 0.25150	0.020 (-0.014, 0.053) 0.24777
Q3	0.040 (0.005, 0.074) 0.02623	0.025 (-0.009, 0.060) 0.14450	0.021 (-0.015, 0.056) 0.25315
Q4	0.081 (0.047, 0.115) <0.00001	0.068 (0.033, 0.103) 0.00015	0.069 (0.031, 0.107) 0.00040
P for trend	<0.00001	0.00022	0.00098

Crude model: we did not adjust other covariates.

Minimally adjusted model: we adjusted SEX; AGE; BMI.

Fully adjusted model: we adjusted SEX; AGE; BMI; HCY; CR; Hypertension; Cystatin C; Antiplatelet drug use; Smoking status; DLP; HDL-C; LDL-C; Alcohol consumption status; Neutrophil count; Albumin; DBP; SBP; triglyceride.

### 3.4 Non-linear relationship between UA and CIMT

**Figure 1:**
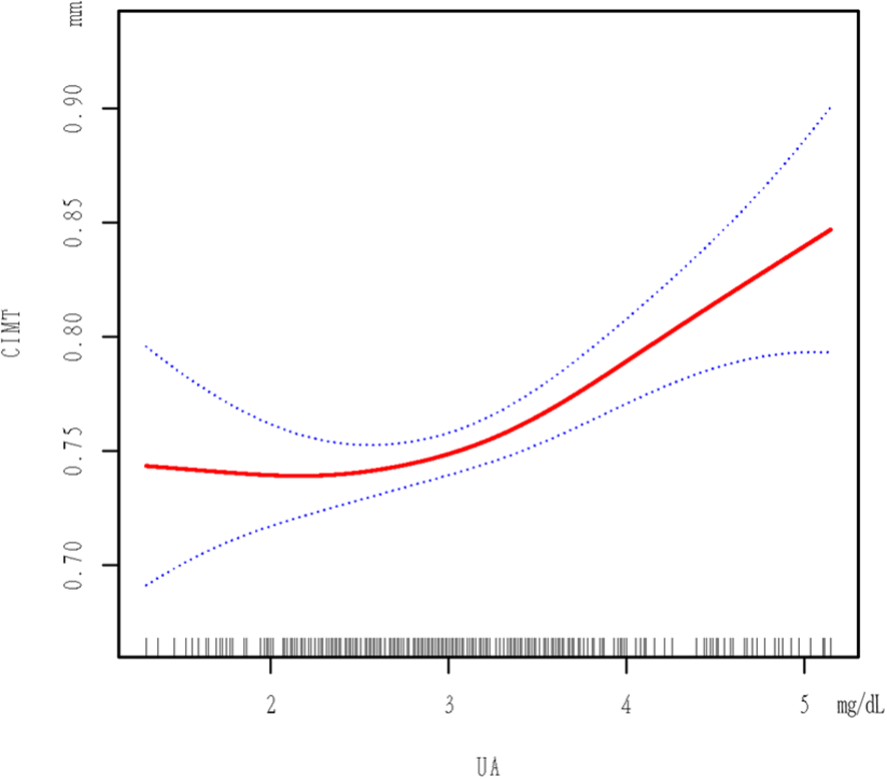
Plot Of Piecewise Linear Regression. The relationship between UA and CIMT. A threshold, nonlinear association between UA and CIMT was found in a generalized additive model (GAM). Solid red line represents the smooth curve fit between variables. Dotted line represents the 95% of confidence interval from the fit. All adjusted for SEX; AGE; BMI; HCY; CR; Hypertension; Cystatin C; Antiplatelet drug use; Smoking status; DLP; HDL-C; LDL-C; Alcohol consumption status; Neutrophil count; Albumin; DBP.

**Figure 1** shows a non-linear relationship between UA and CIMT in the smoothed curve plot, after adjusting for the mentioned confounding factors. **Table 4** revealed the recognition of a pivotal moment in the correlation between UA levels and CIMT. When UA is <3.15 mg/dL, the relationship is not statistically significant (p = 0.9240). Nevertheless, in the UA level range from 3.15 to 5.148 mg/dL, there is a notable and meaningful connection between UA and CIMT, which varies depending on the dosage (adjusted β = 0.061; 95% CI = 0.031-0.090, p < 0.0001).

**Table 4:** The independent association between UA and CIMT by multivariate piecewise linear regression.

Inflection Point of UA (mg/dL)	Effect Size (β)	95% CI	P-value
<3.15	-0.002	(-0.033, 0.030)	0.9240
≥3.15	0.061	(0.031, 0.090)	<0.0001

we adjusted SEX; AGE; BMI; HCY; CR; Hypertension; Cystatin C; Antiplatelet drug use; Smoking status; DLP; HDL-C; LDL-C; Alcohol consumption status; Neutrophil count; Albumin; DBP; SBP; triglyceride.

### 3.5 *post hoc* power analysis

A *post hoc* power analysis was conducted to evaluate the achieved statistical power based on the observed effect size in the multivariate piecewise linear regression model. Power was computed using R (version 4.4.1) with parameters obtained from the primary analysis, assuming a significance level of 0.05 and a critical power threshold of 0.80. The *post hoc* power analysis was conducted using an effect size of 0.0634 and degrees of freedom (19, 320). The estimated power was found to be 0.80.

## 4 Discussion

Elevated levels of UA in the human body have been associated with multiple pathological processes. ​Epidemiological studies consistently identify hyperuricemia as an independent risk factor for cardiovascular events. For instance, Bos et al. demonstrated that hyperuricemia independently predicts myocardial infarction and stroke (22). ​Epidemiological studies consistently identify hyperuricemia as an independent risk factor for cardiovascular events. For instance, Bos et al. demonstrated that hyperuricemia independently predicts myocardial infarction and stroke (23).Research conducted on endothelial cells from the human umbilical vein discovered that elevated levels of UA trigger oxidative stress and inflammation by impacting the signaling pathway of HMGB1/RAGE, the pathway of NF-κB, the activation of the renin-angiotensin system, the reduction of NO, and the expression of inflammatory cytokines (24–26). These mechanisms collectively promote endothelial dysfunction and vascular remodeling, accelerating atherosclerosis.

In our study involving a healthy check-up population, we identified an independent correlation between UA and increased CIMT (β = 0.037; 95% CI = 0.021-0.052, p < 0.00001).The correlation remained significant even after accounting for other variables (β = 0.033; 95% CI = 0.015-0.050, p = 0.00032).The association between increased UA from Q1 to Q4 and CIMT was significant in both minimally adjusted and fully adjusted models (**Table 3**). Significantly, we noticed an inverse correlation between UA and CIMT, exhibiting a critical threshold at 3.15 mg/dL. Below this threshold, UA levels were not statistically associated with CIMT, while above this threshold, a significant positive correlation was observed, suggesting a hormesis phenomenon.

Subsequent analysis using smoothing functions and segmented linear regression models confirmed the threshold effect at 3.15 mg/dL. According to our research, there is a nonlinear correlation between UA and CIMT in the population undergoing regular health check-ups, with a critical value at 3.15 mg/dL. There is no significant correlation between CIMT and UA when UA is less than 3.15 mg/dL, but a significant positive correlation with CIMT is observed when UA is 3.15 mg/dL or higher. Reducing uric acid through treatment can help alleviate factors that cause arterial inflammation and the formation of neointimal lesions in a mouse model induced with carotid atherosclerosis (27).To reduce the risk of increased CIMT, it is suggested that individuals without symptoms should maintain UA levels below 3.15 mg/dL, taking into account the potential advantages of prolonged non-bisphosphonate therapy in preventing arterial stiffness caused by hyperuricemia (28).

The innovation of this study is primarily reflected in the following aspects:​Revealing the nonlinear relationship between UA and CIMT: While previous studies have investigated the association between hyperuricemia and atherosclerosis, few have systematically analyzed the nonlinear relationship between uric acid (UA) and carotid intima-media thickness (CIMT). By employing generalized additive models (GAMs) and piecewise linear regression models, we successfully demonstrated this nonlinear relationship, overcoming the limitations of traditional linear models. Furthermore, a threshold effect of UA levels was identified, providing a more precise reference for clinical intervention.​Highlighting the potential of UA as an early biomarker for arteriosclerosis: Through our analysis, we observed a significant association between elevated UA levels and increased CIMT, particularly when UA concentrations exceeded 3.15 mg/dL. These findings provide a theoretical foundation for the potential application of UA as a biomarker in early arteriosclerosis screening. This discovery may offer new directions for the prevention and intervention of early-stage cardiovascular and cerebrovascular diseases.

Despite these findings, our study has certain limitations. Initially, as a cross-sectional study, it solely illustrates a non-linear correlation between UA and CIMT and cannot establish causation, necessitating prospective studies for verification. Secondly, averaging the measurements of CIMT at six locations on both sides of the neck may not fully reflect the thickness at other locations if there is severe thickening beyond these measured locations. Thirdly, due to collinearity, the use of statin drugs, total cholesterol, and estimated glomerular filtration rate were not adequately adjusted and may influence the results. Additionally, our study population had a high proportion of individuals over 60 years old (67.73%), possibly influenced by factors such as social, economic, and family values, making them more willing to undergo health check-ups. In conclusion, since this study was conducted retrospectively on a population undergoing routine health check-ups, the findings may not be applicable to individuals suffering from different medical conditions.

## Data Availability

The raw data supporting the conclusions of this article will be made available by the authors, without undue reservation.
